# Increase in Blood Levels of Growth Factors Involved in the Neuroplasticity Process by Using an Extremely Low Frequency Electromagnetic Field in Post-stroke Patients

**DOI:** 10.3389/fnagi.2018.00294

**Published:** 2018-09-26

**Authors:** Natalia Cichoń, Michał Bijak, Piotr Czarny, Elżbieta Miller, Ewelina Synowiec, Tomasz Sliwinski, Joanna Saluk-Bijak

**Affiliations:** ^1^Department of General Biochemistry, Faculty of Biology and Environmental Protection, University of Lodz, Łódź, Poland; ^2^Department of Medical Biochemistry, Medical University of Lodz, Łódź, Poland; ^3^Department of Physical Medicine, Medical University of Lodz, Łódź, Poland; ^4^Neurorehabilitation Ward, III General Hospital in Lodz, Łódź, Poland; ^5^Laboratory of Medical Genetics, Department of Molecular Genetics, Faculty of Biology and Environmental Protection, University of Lodz, Łódź, Poland

**Keywords:** extremely low-frequency electromagnetic field, neuroplasticity, brain-derived neurotrophic factor, stroke, rehabilitation

## Abstract

**Background:** Neuroplasticity ensures the improvement of functional status in patients after stroke. The aim of this study was to evaluate the effect of extremely low-frequency electromagnetic field therapy (ELF-EMF) on brain plasticity in the rehabilitation of patients after stroke.

**Methods:** Forty-eight patients were divided into two groups underwent the same rehabilitation program, but in the study group, the patients additionally were exposed to a standard series of 10 ELF-EMF treatments. To determine the level of neuroplasticity, we measured the plasma level of the brain-derived neurotrophic factor (BDNF), the vascular-endothelial growth factor, as well as BDNF mRNA expression. Additionally, we determined the molecule levels for hepatocyte growth factor, stem cell factor, stromal cell-derived factor 1α, nerve growth factor β, and leukemia inhibitory factor, using 5plex cytokine panel in plasma. After 4 weeks, during which patients had undergone neurorehabilitation and neurological examinations, we assessed functional recovery using the Barthel Index, Mini-Mental State Examination (MMSE), Geriatric Depression Scale, National Institutes of Health Stroke Scale (NIHSS), and the modified Rankin Scale (mRS).

**Results:** We observed that ELF-EMF significantly increased growth factors and cytokine levels involved in neuroplasticity, as well as promoted an enhancement of functional recovery in post-stroke patients. Additionally, we presented evidence that these effects could be related to the increase of gene expression on the mRNA level. Moreover, a change of BDNF plasma level was positively correlated with the Barthel Index, MMSE, and negatively correlated with GDS.

**Conclusion:** Extremely low-frequency electromagnetic field therapy improves the effectiveness of rehabilitation of post-stroke patients by improving neuroplasticity processes.

## Introduction

Regenerative processes within the brain tissue are limited and regulated by tissue environmental properties, which are affected by changes in the physiology of the organism (Hagg, 2009). Neurotrophic factors affect neurogenesis through the condition of the growth of new neurons and the survival of existing ones (Hylin et al., 2017). Traditionally, neurotrophic factors are divided into three protein families: the classic neurotrophin, ligands of Glial Cell Derived Neurotrophic Factor (GDNF), and neuropoietic cytokines. Neurotrophins are synthesized and secreted by nerve cells in the brain and spinal cord, and the cells of dependent tissue (Hagg, 2009; Lindholm and Saarma, 2010).

Compensatory plasticity of the damaged brain is a completely different process from the plasticity occurring in a normally functioning, healthy brain. This process is initiated under critical conditions: in interactions with oedema, inflammation, apoptosis, metabolic disturbances, and fiber degeneration. It starts immediately after an ischaemic event (Liguz-Lecznar and Kossut, 2013). Moreover, neuroplasticity consists of strengthening the existing nerve pathways and then establishing new connections. Existing, but weaker connections between brain centers undergo activation (Martin et al., 2017). As a result, the defective function can be restored partially or completely, because other cortical or subcortical structures will take over the function of the damaged area. In the brain in animal models, synaptogenesis was found in the area adjacent to tissue damaged by the stroke, and also in regions of the undammed hemisphere (Law et al., 2017).

Neuroplasticity is closely related to neurogenesis, wherein fully functioning neuronal cells are generated, resulting from the differentiation of neuronal stem cells (NSCs) present in the adult, fully formed brain. NSCs are characterized by the ability of indefinite mitotic division, potential divisions, and to differentiate into the appropriate morphological phenotype (Klein et al., 2016). The process of neurogenesis occurs in certain brain structures throughout life, nevertheless the rate of proliferation and the ability of the newly formed neurons to survive to reduce with age. Nerve cells are generated in brain regions responsible for learning, memory, and reception of olfactory sensation, primarily in the sub-ventricular zone (SVZ) and the subgranular zone (SGZ), and also in the migration of emergent neuroblasts toward injury (Zelentsova-Levytskyi et al., 2017). Neurogenesis is regulated by many factors, including neurotrophins, growth factors, hormones, neurotransmitters, and microenvironmental factors (Aimone et al., 2014).

Physical therapy, including the use of extremely low-frequency electromagnetic field (ELF-EMF) therapy, is beneficial in restoring the patients after stroke. ELF-EMF demonstrates anti-inflammatory, regenerative, analgesic, and osteogenic action. Moreover, ELF-EMF promotes cell proliferation, protein synthesis, ion transport, and changes in cellular signal transmission (Li, 2017). Our previous studies have shown that ELF-EMF therapy reduces oxidative stress during rehabilitation of post-acute stroke patients (Cichoń et al., 2017a, 2018). Additionally, our recent research, for the first time focused on the effect of ELF-EMF on the potential factors of brain plasticity, indicate that ELF-EMF therapy increases the generation and metabolism of NO – neurotransmitter regulating neurogenesis, and synaptic plasticity (Cichoń et al., 2017b). As part of the broadening the examined issue, the present analyses are a continuation of the parameter evaluation of the same group of post-stroke patients but currently relate to changes in blood levels of growth factors involved in the neuroplasticity process, induced by ELF-EMF therapy. We selected two significant growth factors for analysis, i.e., brain-derived neurotrophic factor (BDNF) and vascular endothelial growth factor (VEGF). BDNF is the most common neurotrophin in the nervous system, and playing an important role as an effective indicator for rehabilitation interventions in relation to brain neuroplasticity improvement (Qiao et al., 2017). VEGF is one of the most important pro-angiogenic factors and is critical for blood vessel growth in the nervous system of vertebrates. VEGF-induced blood vessel growth may be essential for nervous tissue regeneration during the recovery process. Moreover, several recent studies demonstrate that VEGF has significant non-vascular functions in the nervous system, and it can be considered as an important agent for promoting neurogenesis, glial growth, and nerve repair (Rosenstein et al., 2010). It is well documented that the cytokine-mediated inflammatory mechanisms within the central nervous system (CNS) contribute to cognitive impairment due to disorders of neurons and glial cells in acute stroke patients. Therefore, we have also chosen a panel of 5 cytokines (HGF, SCF, SDF-1α, β-NGF, and LIF) simultaneous measured using Bio-Plex System, which may be the important factors involved in the neurochemical features of brain tissue damage and repair.

## Materials and Methods

### Blood Sample Collection

Blood samples were taken twice: before and after a standard ten sessions of therapy (with an interval of 14 days). They were collected into CPDA_1_ containing tubes (Sarstedt, Nümbrecht, Germany). For analysis of mRNA expression, a portion of the sample was frozen at -80°C immediately upon collection. The rest of the samples were centrifuged (15 min at 1,500 *g*) at 25°C, to isolate the plasma. All blood samples were collected at the same time of day (between 7 am and 9 am), under conditions of dietary fasting, and stored using the same protocol.

### Subject Presentation

Post-stroke patients with moderate stroke severity (*n* = 48) were recruited to the study, and were then randomly divided into a study group [ELF-EMF (*n* = 25; NIHSS scores of 4.9 ± 3.1; aged 48.0 ± 8.0)], and a control group [non-ELF-EMF (*n* = 23; NIHSS scores of 5.4 ± 2.9; aged 44.8 ± 7.7)]. Clinical and demographic characteristics are shown in **Table 1**. The same patients were enrolment to our previous study (Cichoń et al., 2017b). Subjects with neurological illness other than stroke, haemorrhagic stroke, chronic or significant acute inflammatory factors, dementia, and/or decreased consciousness in their medical pre-stroke history, were all excluded. The patients had undergone neurorehabilitation as well as internal and neurological examinations, for 4 weeks in Neurorehabilitation Ward III of the General Hospital in Łódź, Poland.

**Table 1:** Demographic characteristics.

	Non-ELF-EMF group	ELF-EMF group	*p*
**Demographics**			
Age [mean ±*SD*]	44.8 ± 7.7	48.0 ± 8.0	0.84
Sex (man) [%]	48 vs. 52	60 vs. 40	0.27
Living alone [%]	32.1	34.2	0.59
**Vascular risk**			
Hypertension [%]	97.3	98.5	0.07
Diabetes [%]	31.4	39.2	0.21
Dyslipidemia [%]	78.8	72.2	0.7
BMI ≥ 30 [%]	21	34	0.78
**Concomitant medications**			
Antidepressants [%]	29	34	0.5
ASA [%]	70	65	0.42
NSAID [%]	25	27	0.8
**Stroke characteristics**			
Weeks since stroke [mean ±*SD*]	3.9 ± 0.6	3.2 ± 0.4	
NIHSS scores [mean ±*SD*]	5.4 ± 2.9	4.9 ± 3.1	
ADL [mean ±*SD*]	8.89 ± 2.87	9.95 ± 2.35	0.22
**Lesion location**			
Anterior [*n*]	3	5	
Posterior [*n*]	7	6	
Intermediate [*n*]	13	14	
**Lesion side**			
Left [*n*]	15	13	
Right [*n*]	8	12	

In both subject groups the same therapeutic program (aerobic exercise 30 min, neurophysiological routines 60 min, and 15 min psychological therapy) was used. Furthermore, ELF-EMF therapy was conducted using a Magnetronic MF10 generator (EiE Elektronika i Elektromedycyna, Otwock, Poland). Both groups were treated for the same amount of time (15 min), but sham exposures were administrated to the non-ELF-EMF subjects. Subjects were excluded from the ELF-EMF group who had electronic and/or metal implants (pacemakers, etc.). ELF-EMF therapy with specific parameters (magnetic induction of 5 mT, 40 Hz, rectangular and bipolar waveforms) was conducted in the ELF-EMF group. The pelvic girdle of the patients was exposed to the electromagnetic field.

### Determination of BDNF Level in Plasma

Plasma samples were diluted ten times (using a diluents buffer) before measurement of BDNF concentration, using a Human BDNF ELISA Kit (Abcam, Cambridge, MA, United States), in accordance with the manufacturer’s protocol. The intensity of the color was measured at 450 nm (Schiavone et al., 2017).

### Determination of *BDNF* Expression in Whole Blood Samples

#### Isolation of RNA and Reverse Transcription

Frozen whole blood samples (-80°C) were lysed using TRI Reagent^®^ (Sigma-Aldrich, St. Louis, MO, United States), after which phase separation was performed. Then, an InviTrap Spin Universal RNA Mini Kit (Stratec Biomedical Systems, Birkenfeld, Germany) was used to purify the RNA-containing aqueous phase. The quantity and purity of RNA were estimated using a Synergy HTX Multi-Mode Microplate Reader, equipped with a Take3 Micro-Volume Plate (BioTek Instruments, Inc., Winooski, VT, United States). RNA samples were diluted to 20 ng/μL and transcribed into cDNA with a High-Capacity cDNA Reverse Transcription Kit (Applied Biosystems^TM^, Waltham, MA, United States). All steps were performed according to the manufacturers’ recommendations.

#### Real-Time PCR

Expression levels of the studied genes were obtained using the following TaqMan probes: Hs02718934_s1 for human *BDNF* gene, and Hs02786624_g1 as an endogenous control, which was a human *GAPDH* gene (Life Technologies, Carlsbad, CA, United States). Real-time PCRs were performed in a CFX96 real-time PCR system (Bio-Rad Laboratories, Hercules, CA, United States) using a TaqMan Universal Master Mix II, without UNG (Life Technologies, Carlsbad, CA, United States). All procedures were performed according to the manufacturers’ protocols. Relative expressions of the studied genes were calculated using the equation 2^-ΔCt^, where ΔCt = Ct_targetgene_ – Ct*_GAPDH_*.

### Determination of VEGF in Plasma

Measurement of VEGF concentration was conducted using a VEGF Human ELISA Kit (Novex^®^ Life Technologies, Carlsbad, CA, United States), according to the manufacturer’s protocol. The intensity of the color was measured at 450 nm (Ling et al., 2015).

### Analysis of Plasma Cytokine Levels

The level of HGF, SCF, SDF-1α, β-NGF, and LIF plasma growth factors were indicated using a Human Cytokine 5-plex assay kit (Bio-Rad, Hercules, CA, United States), on a Bio-Plex^®^ 200 system (Bio-Rad, Hercules, CA, United States). Growth factors were measured in accordance with the manufacturer’s protocol (Zanotta et al., 2016).

### Clinical Parameters Determination

The stroke-related neurologic deficit was measured using The National Institutes of Health Stroke Scale (NIHSS). Functional status was evaluated using the Barthel Index of Activities of Daily Living (ADL) and modified Rankin Scale (mRS), and cognitive status using the Mini-Mental State Examination (MMSE). Depression, the most common affective complication after stroke, was estimated using the Geriatric Depression Scale (GDS) (Cichoń et al., 2017a). The NIHSS, ADL, MMSE, GDS, and mRS were conducted either on the same day as the blood sampling or the afternoon before, in both groups.

### Data Analysis

All experiments were performed in duplicate and calculated as mean values. For all subjects, the values of parameters before their treatments were used as the output value (100%). Data from the experiments performed on these same subjects after appropriate treatments were expressed as a percentage of the output value. Values obtained in this way were expressed as mean ± SD.

The all statistical analyses were performed using Stats Direct statistical software v.2.7.2. To avoid committing a type 1 error statistical analysis was performed using multiple comparison methods. First, the Shapiro–Wilk test was used to assess normal distribution of variables. Next, the results were analyzed for equality of variance using Levene’s test. The significance of the differences between the values was analyzed using ANOVA, followed by Tukey’s range test for multiple comparisons (for data with normal distribution and equality of variance) or non-parametric the Kruskal–Wallis test (when variables had other than normal distribution or had no equality of variance) (Bijak et al., 2012, 2013).

Additionally, we performed a correlation analysis between the changes in both experimental and clinical parameters. For these analyses, a Spearman’s rank correlation was used, with the Spearman’s rank correlation coefficient and the probability of correlation designated. For all analyses, a level of *p* < 0.05 was accepted as statistically significant.

## Results

**FIGURE 1:**
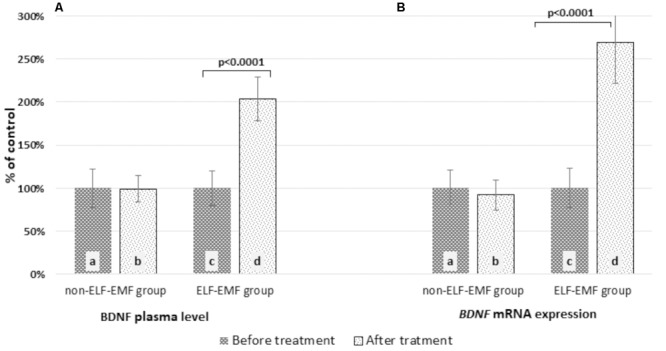
Comparison of the BDNF level obtained from the ELF-EMF group vs. the non-ELF-EMF group. **(A)** BDNF plasma concentration. Statistical significance between ELF-EMF and non-ELF-EMF groups: b vs. d: *p* < 0.0001. **(B)** *BDNF* mRNA expression. Statistical significance between ELF-EMF and non-ELF-EMF groups: b vs. d: *p* < 0.0001.

**FIGURE 2:**
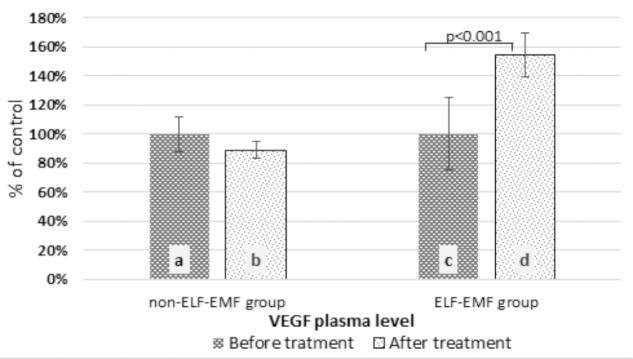
Comparison of the plasma VEGF level obtained from the ELF-EMF group vs. the non-ELF-EMF group. Statistical significance between ELF-EMF and non-ELF-EMF groups: b vs. d: *p* < 0.001.

**FIGURE 3:**
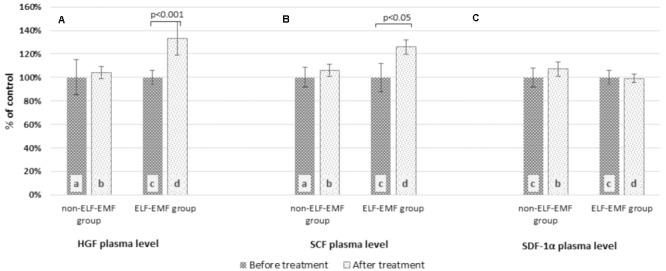
Comparison of the plasma cytokine profile obtained from the ELF-EMF group vs the non-ELF-EMF group. **(A)** HGF plasma level. Statistical significance between ELF-EMF and non-ELF-EMF groups: b vs. d: *p* < 0.01. **(B)** SCF plasma level. Statistical significance between ELF-EMF and non-ELF-EMF groups: b vs. d: *p* < 0.05. **(C)** SDF-1α plasma level.

In our comparative analysis, we demonstrated the effect of ELF-EMF therapy on various neurotrophic factors. Particularly relevant findings relate to the level of BDNF as the most prevalent growth factor in the CNS, which is essential for the development of CNS and neuronal plasticity. The plasma level of BDNF in the ELF-EMF group after ten sessions of rehabilitation was significantly higher compared to the non-ELF-EMF group (*p* < 0.0001). The increase of the BDNF level in the ELF-EMF group was about 200% (*p* < 0.0001), while in the non-ELF-EMF group it was comparable (*p* > 0.05) (**Figure 1A**). We also evaluated the effect of ELF-EMF on gene expression in the whole blood samples of *BDNF*. We demonstrated that after ELF-EMF therapy, expression of *BDNF* increased about 195% (*p* < 0.0001), while in the non-ELF-EMF group it did not change (**Figure 1B**). Because of the crucial role of BDNF participates in the formation of appropriate synaptic connections in the brain, it seems that ELF-EMF may serve as a therapeutic treatment to improve the neuroplasticity after stroke. Moreover, as proved to be significant differences in the level of VEGF caused by ELF-EMF application. VEGF is crucial for cross-talk between the cardiovascular and nervous systems, which is particularly important in the case of brain stroke that damages both blood vessels and nervous tissue. After treatment, the VEGF plasma concentration in the ELF-EMF group increased about 50% (*p* < 0.001), but in the non-ELF-EMF group it remained unchanged (*p* > 0.05) (**Figure 2**). The raw data of both BDNF and VEGF plasma level has been shown in **Table 2**). We also assessed five cytokine concentrations in plasma using the Luminex platform. Two of these (βNGF and LIF) presented as out of range, both before and after treatment (<2.57 pg/ml and 1.92 pg/ml, respectively) (**Table 3**). After treatments, hepatocyte growth factor (HGF) and stem cell factor (SCF) levels in plasma were elevated in the ELF-EMF group (*p* < 0.01 and *p* < 0.05, respectively), but the SDF-1α level was comparable in both groups (*p* > 0.05) (**Table 3** and **Figure 3**). HGF is another (like VEGF) angiogenic factor that also produces neurotrophic effects in CNS. HGF plays pivotal roles in the nervous system during nerve regeneration process, by acting on neuronal or non-neuronal cells. HGF concentration after ELF-EMF therapy increased about 35% (*p* < 0.01), and in the non-ELF-EMF group remained unchanged (*p* > 0.05) (**Figure 3A**). SCF is a cytokine belonging to the control factors of the differentiation of stem cells to neurons and glia. Given the capacity of SCF to induction of regenerate of cells lost through brain injury, this cytokine seems particularly essential for the course of neuroplasticity processes. SCF level in the ELF-EMF group was higher by about 25% after treatment (*p* < 0.05), and in the non-ELF-EMF group was unchanged (*p* > 0.05) (**Figure 3B**). After therapy, the change of SDF-1α concentration was low and not statistically significant in either group (**Table 3** and **Figure 3C**).

**Table 2:** Plasma levels (in pg/ml) of BDNF and VEGF measured before and after treatment in ELF-EMF and non-ELF-EMF groups.

		Non-ELF-EMF group	ELF-EMF group
BDNF [pg/ml]	Before treatment	25.57	23.68
	After treatment	23.31	36.36
	*p*	>0.05	<0.001
VEGF [pg/ml]	Before treatment	37.67	30.75
	After treatment	34.79	46.29
	*p*	>0.05	<0.001

**Table 3:** Cytokine plasma profile.

		Non-ELF-EMF group	ELF-EMF group
HGF [pg/ml]	Before treatment	369.55	274.43
	After treatment	390.83	366.62
	*p*	>0.05	<0.001
SCF [pg/ml]	Before treatment	96.21	85.66
	After treatment	98.68	105.29
	*p*	>0.05	<0.05
SDF-1α [pg/ml]	Before treatment	105.55	122.45
	After treatment	108.65	120.40
	*p*	>0.05	>0.05
βNGF [pg/ml]	Before treatment	<2.57	<2.57
	After treatment	<2.57	<2.57
LIF [pg/ml]	Before treatment	<1.92	<1.92
	After treatment	<1.92	<1.92

**FIGURE 4:**
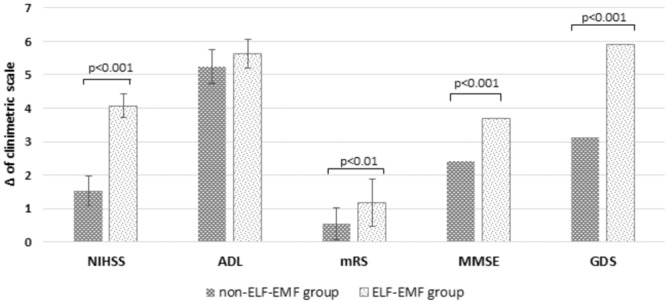
Clinical parameters NIHSS, ADL, mRS, MMSE, and GDS as measured in the study vs. the control group. Data is shown here as a delta of scores obtained before and after the standard series of treatments (Δ NIHSS = the decline of NIHSS; ΔADL = the gain of ADL; Δ mRS = the decline of mRS; Δ MMSE = the gain of MMSE; Δ GDS = the decline of GDS).

Additionally, we estimated the clinical status of patients using NIHSS, ADL, mRS, MMSE, and the GDS in both groups. Stroke-related neurologic deficit estimated using NIHSS in ELF-EMF group decreased about 65% more than in non-ELF-EMF group (*p* < 0.001) (**Figure 4**). Functional status assessed by ADL in both groups increased (*p* < 0.001 and *p* < 0.001), but ΔADL in both groups was comparable (*p* > 0.05), whereas assessed by mRS decreased in both group, and decline in ELF-EMF group was more about 50% than in non-ELF-EMF group (*p* < 0.01) (**Figure 4**). A better improvement after ELF-EMF therapy was observed in cognitive impairment estimated by MMSE, with about a 35% higher growth (**Figure 4**). Depressive syndrome measured in GDS decreased significantly, while ΔGDS gained about 45% better results in the ELF-EMF group than the non-ELF-EMF group (**Figure 4**).

**FIGURE 5:**
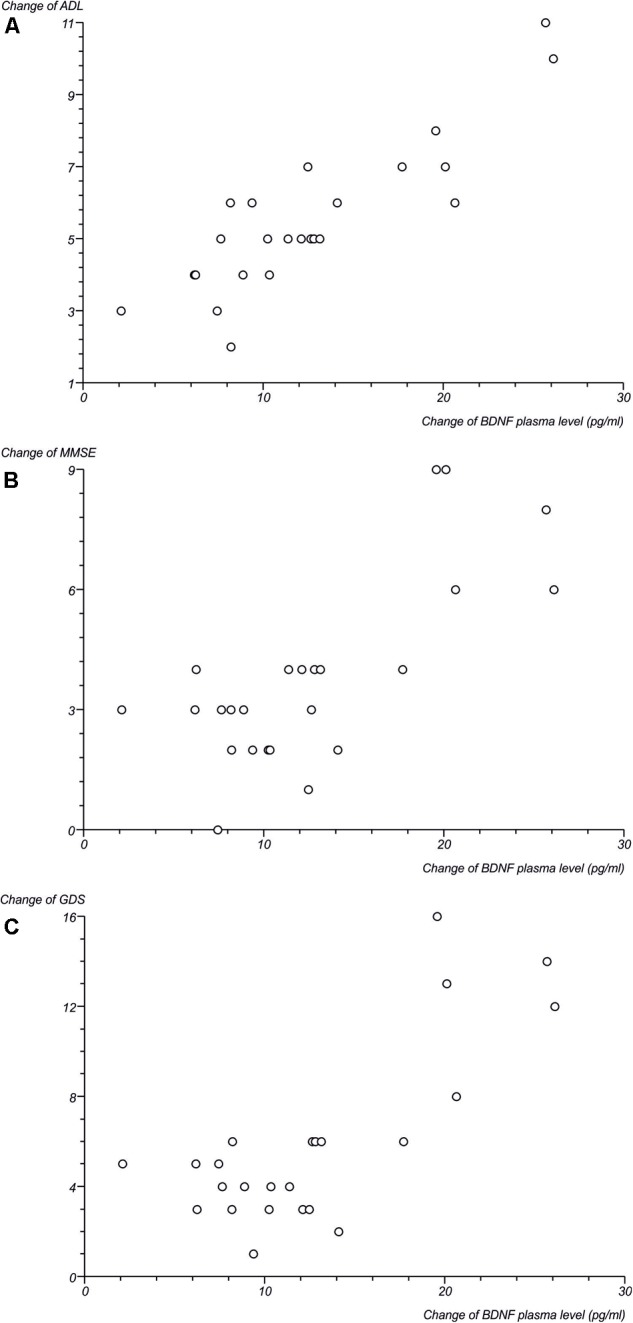
Scatterplots presenting correlation between changes of BDNF plasma level (in pg/ml) in ELF-EMF group and **(A)** changes of ADL; **(B)** changes of MMSE; **(C)** changes of GDS.

Subsequently, we performed a correlation analysis between the plasma level of the most important neurotrophic factor, BDNF, and clinical parameters. Correlation parameters indicated a significant positive correlation between changes (Δ) of BDNF plasma level and ΔADL, as well as between ΔBNDF and ΔMMSE, and a negative correlation between ΔBNDF and ΔGDS (**Figure 5**). The detailed process of these relationships, which confirms the correlation and the numeric data shown, is presented in **Table 3**.

## Discussion

Post-stroke rehabilitation is intended to restore the patient to a healthy condition and return them to a functional state, as prior to their illness, or allow them to adapt and achieve an optimal level of independence. Active post-stroke therapy should be begun as soon as possible, immediately upon stabilization of the general medical state. The rehabilitation process should be continued until obtaining a good result on an actual improvement index, which should also include cognitive disorders and behavioral changes (Jia et al., 2017).

Ischaemic stroke and other acute CNS injury indicates an increase of neuronal progenitor’s proliferation in the SVZ zone identifying the lesion area and neuroblast migration to the ischaemic area within the striatum and cortex (Hu et al., 2013; Liu et al., 2015b; Choi et al., 2017).

In current research on brain plasticity processes, particular attention has been focused on BDNF an activator of various signaling pathways involved in regulation of neurogenesis and survival of neurons. The BDNF function may also be related to the formation and maintenance of dendritic spines and dendrites, as well as regulation of synaptic function during long-term potentiation, learning, and memory process (Greenberg et al., 2009). The source of BDNF is an active microglial, as well as the endothelium and neurons (Gomes et al., 2012). BDNF is involved in the regulation of neurogenesis in the SVZ zone and the migration of progenitor cells from the SVZ to the damaged striatum (Bathina and Das, 2015). BDNF infusions into lateral ventricles cause duplication of a number of neurons in the olfactory bulb region and production of synaptic connections (Bath and Lee, 2010).

In this study, we showed for the first time that ELF-EMF increased BDNF concentration (**Figure 1A**), as well as *BDNF* mRNA expression *in vivo* in humans (**Figure 1B**). Our results coincide with those of studies conducted by Di Loreto et al. (2009). They investigated changes in the expression profile of cytokine and the growth factor profile in rat cortical neurons with exposure to ELF-EMF (50 Hz, 0.1 and 1 mT) during maturation of cells. They observed that ELF-EMF exposure induced an increased mRNA and protein expression of BNDF and its receptor. As such, they found that ELF-EMF caused up-regulation in neurons (Di Loreto et al., 2009).

The regenerative ability of ELF-EMF has been confirmed by Hei et al. (2016) They observed that pulsating electromagnetic fields (50 Hz, 1 mT) caused increased *BDNF* gene expression in immortalized rat Schwann cells. They suggested that the electromagnetic field improved regeneration of peripheral nerves by enhancing proliferation of cells, and *BDNF* and *S100* gene expression (Hei et al., 2016). Similar evidence was provided by Urnukhsaikhan et al. (2017). The increase of BDNF, protein level after EMF treatment, confirmed the neuroprotective action of EMF in mice during recovery process after stroke (Urnukhsaikhan et al., 2017).

The probable mechanism of increase of *BDNF* mRNA expression after EMF treatment was explained by Li et al. (2014). They investigated L-type voltage-gated calcium channels-and Erk-dependent signaling pathways and sampled *BNDF* mRNA expression in cultured dorsal root ganglion neurons (Li et al., 2014). Sun et al. (2016) investigated membrane capacitance and calcium influx in the calyx of Held. They certified the significance of the effect of ELF-EMF on plasticity and synaptic transmission by facilitation of synaptic plasticity in a calcium-dependent manner, and vesicle endocytosis. Exposition of ELF-EMF causes increases in the impact of calcium influx on the enhancement of calcium channel expression at the presynaptic nerve terminal (Sun et al., 2016).

The primary function of VEGF is a pro-angiogenic action, but there is much evidence of its neurotrophic and neuroprotective effect, both on the central and peripheral nervous system (Saban et al., 2011; Yao et al., 2016; Shahhoseini and Bigdeli, 2017). VEGF generated by ependymal cells activates and enhances neuronal precursor proliferation and growth in the SCZ and SGZ zones (Chodobski et al., 2003). Moreover, VEGF enhances astrocytes proliferation and migration (Lenzer-Fanara et al., 2017), as well as stimulates growth and survival of Schwann cells after hypoxia (Cattin et al., 2015). Its neurogenesis effect is assessed by stimulation of the endothelium to release neurotrophic factors (Feng et al., 2012).

In the current study, we observed that the VEGF plasma level increased in the group exposed to ELF-EMF (**Figure 2**). However, our results are compatible with studies conducted by Delle Monache et al. (2008), who suggested ELF-EMF’s impact on *in vitro* modulation of endothelial functions through VEGF-dependent signal transduction pathways. Liu et al. (2015a) estimated the effect of a pulsating electromagnetic field on neurotrophic genes’ expression and proliferation in Schwann cells in rats. They observed that EMF increased both protein levels and the gene expression of VEGF, BDNF, and GDNF. They thereby confirm that EMF therapy improves nerve regeneration (Liu et al., 2015a).

We also measured the cytokine plasma levels involved in neuroplasticity processes: HGF, SCF, SDF-1α, β-NGF, and LIF. We observed that after the application of ELF-EMF, hepatocyte growth factor increased (**Table 3** and **Figure 3A**). HGF is expressed in many different tissues, including the brain (Sharma, 2010). HGF by cMet receptor impacts morphogenesis, cell motility, and proliferation of neuronal and non-neuronal tissues. It also activates the migration and proliferation of the progenitors of oligodendrocyte, Schwann cells, as well as intensifying the differentiation and survival of hippocampal, cortical, and midbrain dopaminergic neurons (Wang et al., 2011). Shang et al. (2011) found that in Wistar rats, after transient middle cerebral artery occlusion (tMCAO), exogenous administration of HGF caused a decrease in infarct size, intensification of synaptogenesis and angiogenesis, and a reduction of scar thickness of the pia mater and glial scar formation (Shang et al., 2011). Similarly, Doeppner et al. (2011) investigated the impact of intrastriatal HGF treatment on the long-term effects of and neurologic recovery and brain injury. They suggested that long-term neuroprotection caused by HGF was associated with enhanced neurovascular remodeling, and maintained recruitment of proliferating cells (Doeppner et al., 2011).

Stem cell factor (SCF) also has an impact on neuroprotection and neurogenesis. It is involved in the development of the cortex, and migration and proliferation of neural progenitor cells. SCF in microglia enhances BNDF and NGF expression and reduces pro-inflammatory cytokine expression. Furthermore, SCF participates in neuron-glia, and neuron-neuron interaction (Benedetti et al., 2016). Liu et al. (2016) estimated the effect of administration of SCF and granulocyte-colony stimulating factor (G-CSF) on the effectiveness of recovery in aged mice after stroke. A similar study was conducted by Cui et al., who investigated the effect of the SCF and G-CSF combination on brain repair, 6 months after cortical injury in transgenic mice. They also suggested that SCF + G-SCF treatment enhanced motor function through vascular and synaptic regeneration (Cui et al., 2016). In this study, we showed that the plasma concentration of SCF increased about 25% in the ELF-EMF group (**Table 3** and **Figure 3B**), and this is consistent with Fan et al. whose evaluated the effect of ELF-EMF (50 Hz, 1 mT) on cytokine production and proliferation of mesenchymal stem cells (MSC) in rats. They found that *SCF* mRNA expression after ELF-EMF increased in comparison to their control group. They suggested that ELF-EMF enhanced the proliferation of MSC, as well as up-regulated haematopoietic growth factor expression (Fan et al., 2015).

Stromal derived factor-1α (SDF-1α) is a chemokine secreted from the endothelium which can induce neuroblast migration from SVZ to the ischemic area in rats (Zhao et al., 2015). Luo et al. (2014) demonstrated the impact of physical exercise on functional recovery by improving migration, differentiation, and proliferation of NSCs in SDF-1α rats after MCAO. In our study, we observed no change in SDF-1α, in both the ELF-EMF and non-ELF-EMF groups (**Table 3** and **Figure 3C**).

Despite nerve growth factor (NGF) being a neurotrophic factor predominantly involved in neuroplasticity (Isaev et al., 2017), in our study, the βNGF level in both groups before and after treatment was out of range (**Table 3**). Similarly, leukemia inhibitory factor (LIF), which is an anti-inflammatory cytokine involved in brain plasticity (Vidal et al., 2013), was out of range (**Table 3**). In research by Sarchielli et al. (2013) the level of these molecules was also below the detection limit.

In our study we suggested that ELF-EMF improved neuroplasticity, which idea is compatible with data from the existing literature (Oda and Koike, 2004; Cuccurazzu et al., 2010; Balassa et al., 2013; Cheng et al., 2015). Cuccurazzu et al. (2010) investigated the effect of ELF-EMF (50Hz, 1mT) on hippocampal neurogenesis in adult mice. They observed that ELF-EMF exposition increased the expression of Mash1, NeuroD2, and Hes1 (pro-neuronal transcription genes), and genes encoding Ca(v)1.2 channel α(1C) subunits, thus promoted neurogenesis in the dentate gyrus (Cuccurazzu et al., 2010). On the other hand, Balassa et al. estimated the impact of long-term ELF-EMF (50 Hz, 3mT) synaptic functions in the developing brain. They found that exposure to ELF-EMF enhanced synaptic plasticity and basic neuronal functions in the brain, both in newborn and fetal rats (Balassa et al., 2013). Oda and Koike examined the effect of ELF-EMF (50 Hz, 0.3 mT) on neuronal apoptosis. They observed that application of ELF-EMF inhibited apoptosis and enhanced the survival of immature cerebellar granule neurons (Oda and Koike, 2004). Moreover, Cheng et al. investigated the impact of ELF-EMF (50 Hz, 0.4 mT) on hippocampal neural progenitor cells from both ischaemic and embryonic brains. They observed that application of ELF-EMF intensified the ability of neural progenitor cells to proliferate in both kinds of the brain (Cheng et al., 2015).

In our study, we also found a relationship between ELF-EMF and the enhancement of the clinical parameters of tested subjects, as well as a correlation between BDNF level and clinical parameters (**Figure 5**). The results we obtained show that the ADL value was comparable in both groups of patients (**Figure 4**) and that there was a significant positive correlation between an increase of BDNF level and ΔADL in the ELF-EMF group (**Figure 5** and **Table 4**). Our findings are compatible with results obtained by Zhang et al. (2017). They evaluated functional recovery and serum BDNF level in post-stroke patients and the relationship between the two. They observed a positive correlation between BNDF level and Barhel Index, and between BNDF level and mRS, which indicated functional status (Zhang et al., 2017). The increase in the MMSE parameter before and after ELF-EMF treatment was about 15% (**Figure 4**), and we proved a significantly positive correlation between ΔBDNF level and ΔMMSE in our study group (**Figure 5** and **Table 4**). A positive correlation between BDNF level and the MMSE scale was previously shown by Belviranli et al. (2016) who investigated the dependence of cognitive parameters (assessed by MMSE) and BDNF plasma level in endurance athletes. Similarity, Levada et al. (2016) observed that after BDNF administration in patients with mild neurocognitive disorders, their MMSE level increased. The decline in parameters on the GDS scale was about 50% greater in the ELF-EMF group, in comparison to the non-ELF-EMF group. We also demonstrated a significant positive correlation between changes of BDNF level and ΔGDS (**Figure 5** and **Table 4**). Importantly, a low BDNF level is associated with post-stroke depression and acute stroke (Yang et al., 2011), and over-expression of BDNF in the hippocampus moderates depression behaviors in post-stroke depressive rats (Chen et al., 2015).

**Table 4:** Correlation coefficient values obtained for the change in BDNF plasma level (ΔBDNF) and parameters of functional status (ADL, MMSE, and GDS) after ELF-EMF treatment.

Δ BDNF plasma level		
ADL	MMSE	GDS
Rho = 0.7720	Rho = 0.5979	Rho = -0.55924
*p* < 0.0001	*p* < 0.01	*p* < 0.01
H1: positive correlation	H1: positive correlation	H1: negative correlation

*The correlation was made using Spearman’s rank correlation method. This table includes Spearman’s rank correlation coefficient (Rho), the probability of correlation (p), and hypotheses verification (H1).*

## Conclusion

ELF-EMF improves functional recovery in stroke patients by improving neuroplasticity processes. Intensifying brain plasticity using ELF-EMF therapy is associated with an increased level of neurotrophic factors, which could be caused by the impact of ELF-EMF on gene expression. We also suggested that the inclusion of ELF-EMF treatment in post-stroke therapy could enhance the effectiveness of the therapy.

## Ethics Statement

This study was approved by the Bioethics Committee of the Faculty of Biology and Environmental Protection of University of Lodz, Poland with Resolution No. 13/KBBN-UŁ/II/2016. All participants gave their written informed consent before participation. The study was executed according to the principles of the Helsinki Declaration.

## Author Contributions

NC was involved in the preparation of the research project, preparation, collection and interpretation of data, as well as preparation of the manuscript. MB was involved in the interpretation of data, statistical analysis, and preparation of the manuscript. PC was involved in the preparation of samples (isolation of RNA) and determination of VEGF. EM was responsible for patient recruitment and was involved in the analysis and interpretation of clinical data. ES was involved in the evaluation of the quality of RNA and preparation of cDNA. TS analyzed and interpreted of obtained data and was responsible for language correction. JS-B was involved in the preparation of the research project, critically revision, analysis and interpretation of the data, and preparation of the manuscript. All authors read and approved the final manuscript.

## Conflict of Interest Statement

The authors declare that the research was conducted in the absence of any commercial or financial relationships that could be construed as a potential conflict of interest. The reviewer EC-J and handling Editor declared their shared affiliation at the time of the review.
